# On the Crush Behavior and Energy Absorption of Sustainable Beverage Cans and Their Polyurethane Foam-Filled Structures: An Experimental Study

**DOI:** 10.3390/ma17112655

**Published:** 2024-05-31

**Authors:** Zelin Wang, Zheng Liu, Yangzuo Liu, Wuning Ma, Zhendong Zhang, Changfang Zhao, Chunhao Yang

**Affiliations:** 1 School of Mechanical Engineering, Nanjing University of Science and Technology, Nanjing 210094, China; wangzelin@njust.edu.cn (Z.W.); liuzheng@njust.edu.cn (Z.L.); liuyangzuo@njust.edu.cn (Y.L.); kkmwn@163.com (W.M.); zzd1157@163.com (Z.Z.); 2 Department of Engineering Mechanics, CNMM and AML, Tsinghua University, Beijing 100084, China

**Keywords:** beverage cans, polyurethane foam, thin-walled structures, crush behavior, energy absorption, crashworthiness

## Abstract

In the pursuit of global energy conservation and emissions reductions, utilizing beverage cans as energy-absorbing components offers potential for a sustainable economy. This study examines the impact of foam filling on the crushing behaviors and energy absorption of various types of beverage cans. Quasi-static compression tests were conducted on five geometrically sized cans filled with three densities of polyurethane foam to study their deformation modes and calculate crashworthiness parameters within the effective stroke. Results show that empty beverage cans have lower energy absorption capacities, and deformation modes become less consistent as can size increases. Higher foam density leads to increased total energy absorption, a slight reduction in the effective compression stroke, and a tendency for specific energy absorption to initially increase and then decrease. Regarding crush behavior, smaller cans transition from a diamond mode to a concertina mode, while larger cans exhibit a columnar bending mode. Next, the coupling effect of energy absorption between foam and cans was analyzed so as to reveal the design method of energy-absorbing components. The specific energy absorption of smaller cans filled with polyurethane foam is superior to that of similar empty cans. These findings provide valuable insights for selecting next-generation sustainable energy absorption structures.

## 1. Introduction

The energy absorption (EA) behavior of tubular thin-walled structures (TWSTs) has been extensively studied over several decades [1,2,3]. These structures are widely used in various fields, such as transportation, railways, shipping, and aerospace, due to their light weight, high strength, and affordability [4,5,6]. The materials used in TWSTs have evolved from traditional metal tubes [7] to fiber-reinforced plastics (FRP) tubes [8,9,10,11] and metal–FRP hybrid tubes [12,13,14]. Similarly, the structural forms have diversified from simple circular cross-sections [15] to quadrilateral [16] and polygonal cross-sections [17], multi-cell tubes [18,19], and bionic structures [20,21,22]. To enhance the crashworthiness of TWSTs, researchers have explored filling them with honeycombs [23,24] and porous materials [25,26]. Moreover, with the advancements in 3D printing technology, lattice structures [27,28,29] have been investigated as potential filling core materials for TWSTs. Experimental and simulation analysis have demonstrated their effective EA capabilities. However, the practical application of bionic multicellular structures and lattice-filled structures manufactured by 3D printing is still limited due to cost and productivity constraints. Currently, circular and square cross-sections are predominantly used in energy-absorbing TWSTs, with honeycomb [30], metal foam [31,32], and polymer foam [33] being the primary filling materials. In addition to solid filling materials, research has also been conducted on the crushing behavior of pressurized tubes filled with liquids and gases. Paquette and Kyriakides [34] conducted axial compression experiments on stainless steel tubes filled with liquids. The experiment revealed that cylinders filled with internal pressure exhibited axially symmetrical wrinkles, while those without internal pressure showed asymmetrical wrinkles. However, it is important to note that the compression strokes in this test were all less than 5%. Similarly, Zhang [35] conducted axial compression tests under internal pressure conditions on three groups of steel cans with diameter-to-thickness ratios ranging from 120 to 200. The presence of gas internal pressure can alter the deformation mode of the metal tube wall. Whether it involves a liquid or gas filling, the primary objective of the design is to achieve adaptive energy absorption in the structure. This involves controlling the internal pressure of the liquid or gas in the metal tank through a pump or valve to modify the energy absorption state of the structure. To achieve this goal, additional components such as pumps, pipelines, sealing components, etc., are necessary. These additional components not only increase the cost of the energy absorber but also restrict the product’s application range. The challenge now lies in realizing the design of low-cost, easy-to-process, and recyclable adaptive energy absorbers [36]. Unlike liquid and gas fillers, porous foam, as a typical solid filler, exhibits a low Poisson’s ratio. When used as a filler in pipe fittings, the filled fittings are less susceptible to rupture. Even in the event of local damage, the internal filler does not leak like liquids or gases and retains the ability to absorb energy. Furthermore, polyurethane foam, as a solid filler, can easily adjust its density through composition control, thereby altering its mechanical properties. This characteristic enhances the energy-absorbing capabilities of the filling structure. Additionally, its low cost and straightforward production process make it a highly advantageous material for energy absorbers. 

With the worsening of environmental problems, there is an increasing focus on the recyclability of materials. Consequently, research on the treatment and recycling of solid waste is gaining more attention [37,38,39]. Among various types of solid waste, metal beverage cans are particularly significant in the aluminum material production cycle. It is projected that the global market value of metal beverage cans will reach US $58.25 billion by 2024 [40]. Bao et al. [41] introduced a laboratory evaluation method for aluminum packaging recycling processes, encompassing collection type, pretreatment methods, yield measurement, and metal quality analysis. Stotz et al. [42] examined the recycling process of British aluminum beverage cans and compared material recovery rates in eight different recycling scenarios. Clearly, metal beverage cans have become a vital component of the circular economy, and extensive efforts have been made to explore alternative uses for discarded beverage cans.

A limited number of articles have recently discussed the use of specific models of beverage cans as energy-absorbing components in research. Palanivelu et al. [43,44] conducted drop-weight impact tests and explosion-loading experiments on empty beverage cans to explore their potential as explosion-proof sacrificial layers in buildings. The study examined the dynamic breakage rules of beverage cans and considered the influence of the inertia of the skin panel on the dynamic crushing behavior during the explosion experiment. Ashrafi et al. [45] focused on beverage cans with polyurethane foam caulking and studied the strengthening effect of pressurized foam on TWSTs. Liu et al. [46] conducted quasi-static and dynamic compression experiments on empty cans and foam-filled cans with a capacity of 330 mL at different compressive loading rates. The experiments showed that the initial peak stress of empty beverage cans increased with the increase in the loading rate. Additionally, the filling of polyurethane foam not only changed the deformation mode of the beverage cans but also significantly enhanced the crashworthiness of the cans. Similarly, Chen et al. [47] conducted axial and lateral crush tests on a steel foam-filled beverage can, discovering that the new composite structure displayed remarkable EA capabilities in both quasi-static and dynamic conditions. This finding enhances the potential for widespread utilization of waste beverage cans as efficient and cost-effective energy absorbers. However, recent research primarily focuses on a specific type of beverage can. In reality, beverage cans come in various sizes and are characterized by TWSTs, with their crashworthiness being influenced by the structural dimensions.

To further explore the potential application of waste beverage cans as energy absorbers, this study selected five common sizes of aluminum beverage cans available on the market and filled them with polyurethane foam of varying densities, and quasi-static compression experiments were conducted to analyze the axial crushing behavior of cans with different sizes and filling densities. Furthermore, the characteristics of EA were calculated and compared to investigate the crush behaviors of beverage cans with varying structures and foam densities. This paper will provide valuable insights into the potential use of beverage cans as sustainable energy-absorbers.

## 2. Materials and Methods

### 2.1. Specimens

**Figure 1:**
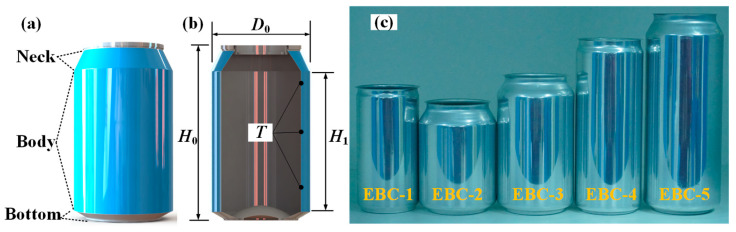
Beverage can specimens. (**a**) Geometric model; (**b**) dimensional parameters; (**c**) five types of empty beverage cans.

The objective of this study is to analyze aluminum beverage cans of five commonly used sizes on the market. These cans are typically used to hold carbonated drinks, juices, and beers. Each beverage can is divided into three parts: the neck, the body, and the bottom, as shown in Figure 1a. The capacities of the five cans are 190 mL, 250 mL, 330 mL, 330 mL, and 500 mL, respectively, and they are named as EBC-1, EBC-2, EBC-3, EBC-4, and EBC-5, where EBC stands for Empty Beverage Can, as depicted in Figure 1c. In this study, the diameter of the can is denoted as *D*_0_, the total height as *H*_0_, and the height of the body as *H*_1_. Since the cans are manufactured using stamping technology, the thickness of the can is not uniformly distributed. Thus, we consider the average thickness, denoted as *T*, at three points: the upper, middle, and lower parts of the can body, as shown in Figure 1b. Thickness data for each measurement point are listed in Table S1 in the supporting materials. Upon visual inspection, it is noted that the necks of EBC-1 and EBC-4 are shorter, while the necks of EBC-2, EBC-3, and EBC-5 are longer. Table 1 provides the geometric parameters of the five samples.

The foam-filled specimens are named according to the FFC-A-B rule. FFC stands for Foam-Filled Cans, where A represents the type of beverage can ranging from EBC-1 to -5. The third digit, B, represents the density of the foam. In this study, three polyurethane foams with varying densities (40 kg/m^3^, 100 kg/m^3^, and 140 kg/m^3^) were prepared. For instance, FFC-1-40 refers to an EBC-1 filled with polyurethane foam that has a density of 40 kg/m^3^.

**Figure 2:**
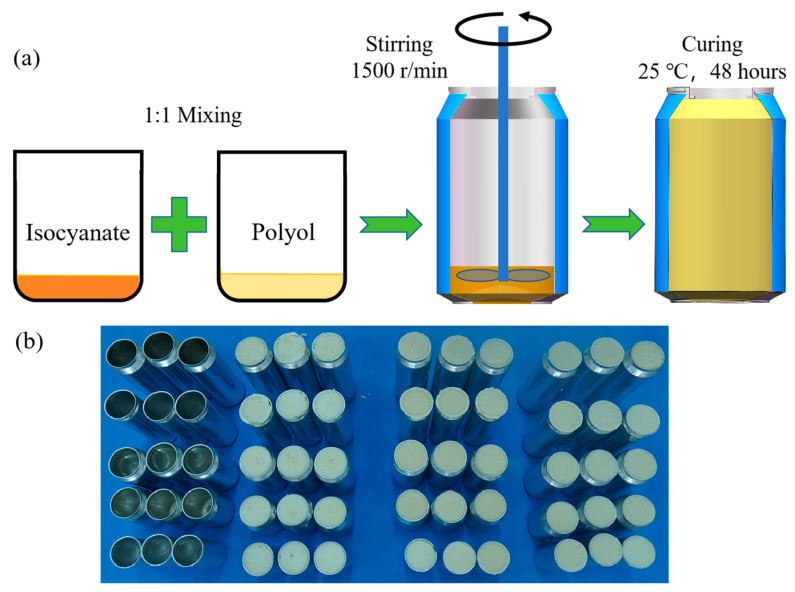
Preparation process and sample of EBCs and FFCs. (**a**) Foaming and filling process of FFCs; (**b**) different types of EBCs and FFCs.

The foam used in the test is polyurethane rigid foam, which exhibits characteristics such as light weight, strong load-bearing capacity, and good weather resistance. The polyurethane foam raw materials utilized in this study were provided by Beijing Hibis Materials Co., Ltd. The sample preparation process is illustrated in Figure 2a. Isocyanate and polyether polyol, the foaming raw materials, are added to the beverage can in a mass ratio of 1:1. A mixer is employed to physically stir the mixture until foaming is complete. Subsequently, the mixture is allowed to mature at room temperature (25 °C) for over 48 h. The excess foam at the mouth of the jar is then trimmed off to finalize the preparation of the energy-absorbing configuration. To ensure test accuracy, three sets of specimens were prepared and tested for each sample. A total of 60 specimens were subjected to compression tests. The prepared specimens are depicted in Figure 2b.

### 2.2. Experimental Methods

**Figure 3:**
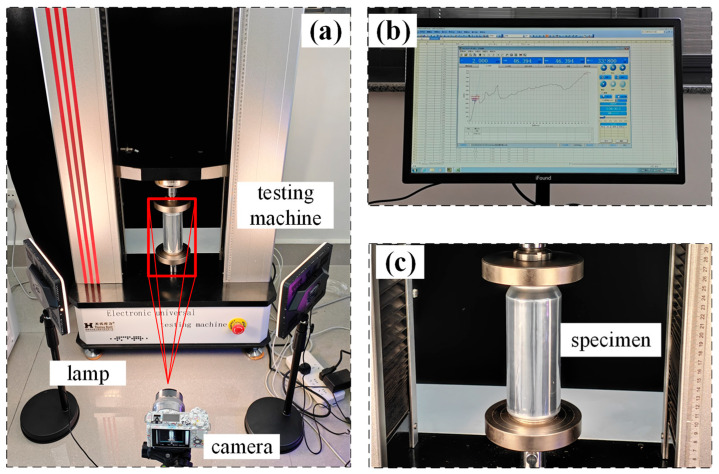
Experimental set-up. (**a**) Compression test system; (**b**) calculation and visualization of test results; (**c**) specimen installation.

Quasi-static axial compression tests were performed on the samples at a compression rate of 5 mm/min using the TSKL-S-20KN universal testing machine (Tinius Kuli Company in Su Zhou, China). The engineering nominal strain ε is used to represent the compression state. It is expressed as Equation (1),

(1) $$\varepsilon =\frac{h}{H_{0}}$$

where *h* represents the compression stroke and *H*_0_ is the total height of beverage cans, as shown in Table 1. The experimental setup is illustrated in Figure 3. The acquisition system records the time, force, and displacement data, while the digital camera captures the crushing process during the test.

### 2.3. Material Properties

**Figure 4:**
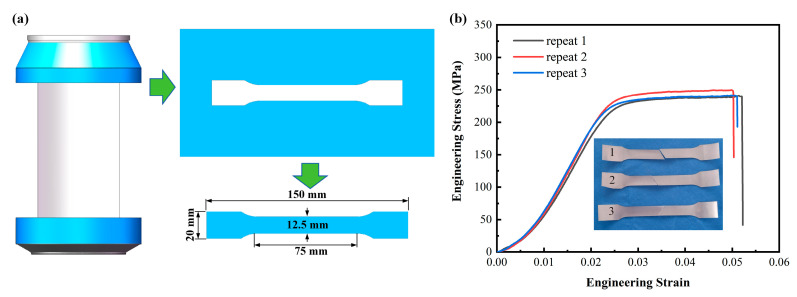
In situ strength testing of beverage can. (**a**) In situ sampling; (**b**) stress-strain curves and failure condition.

In contrast to traditional metal thin-walled tubes, the aluminum cans used in the experiment were manufactured through the deep drawing process [48]. These cans were made from aluminum alloy 3104-H19, with the interior coated with water-based epoxy resin. Due to the significant deformation that occurs during the deep drawing process, defects and uneven dimensions are inevitable. Therefore, the can body is cut in situ and the mechanical parameters of the aluminum alloy material are calibrated. The specimen the test standard followed is ISO 6892-1, with a tensile rate of 5 mm/min, repeated three times. Figure 4 illustrates the preparation process and test results of aluminum alloy samples taken from a beverage can. Under tensile loading, the engineering stress-strain curves exhibit an obvious elastic-plastic mechanical behavior. 

**Figure 5:**
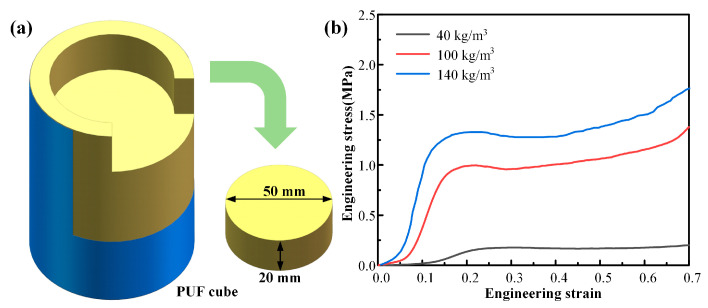
Mechanical properties of polyurethane foams. (**a**) In situ sample and specimen dimensions; (**b**) engineering stress-strain curves of different foam densities.

In order to investigate the contribution of foam to the compressive performance of composite beverage cans, three polyurethane foams with a diameter of 50 mm and a height of 20 mm were obtained through in situ sampling. Compression experiments were conducted following the ASTM C365/C365M standard. The test was repeated 2 times for each density of polyurethane foam specimen. The dimensions of the specimens are shown in Figure 5a. The stress-strain curves of the samples are depicted in Figure 5b. It can be seen that as the density increases, the Young’s modulus, strength, and stress plateau increase, and the load platform for EA is clearly longer.

## 3. Results and Discussion

### 3.1. Crushing Behavior of Empty Beverage Cans

**Figure 6:**
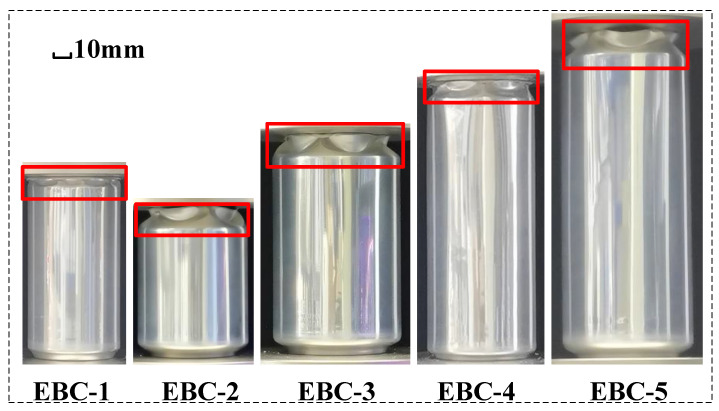
Initial buckling of the neck of each type of EBC.

Previous studies have shown that the deformation patterns of metallic round tubes are influenced by the diameter “*D*”, length “*H*”, and thickness “*t*” [49]. These deformation modes are typically categorized into symmetric modes (concertina-like shapes), asymmetric modes (diamond-like shapes), irregular modes, and Euler bending modes. In the experiment, the length-to-diameter ratio of metal beverage cans ranged from 1.39 to 2.53, while the diameter-to-thickness ratio ranged from 346.41 to 589.29. The inherent concave structural characteristics of the necks of the beverage cans lead to initial buckling typically occurring at the neck. During testing, a circumferential concave triangular fold typically appears at the notch of the neck, as illustrated in Figure 6.

**Figure 7:**
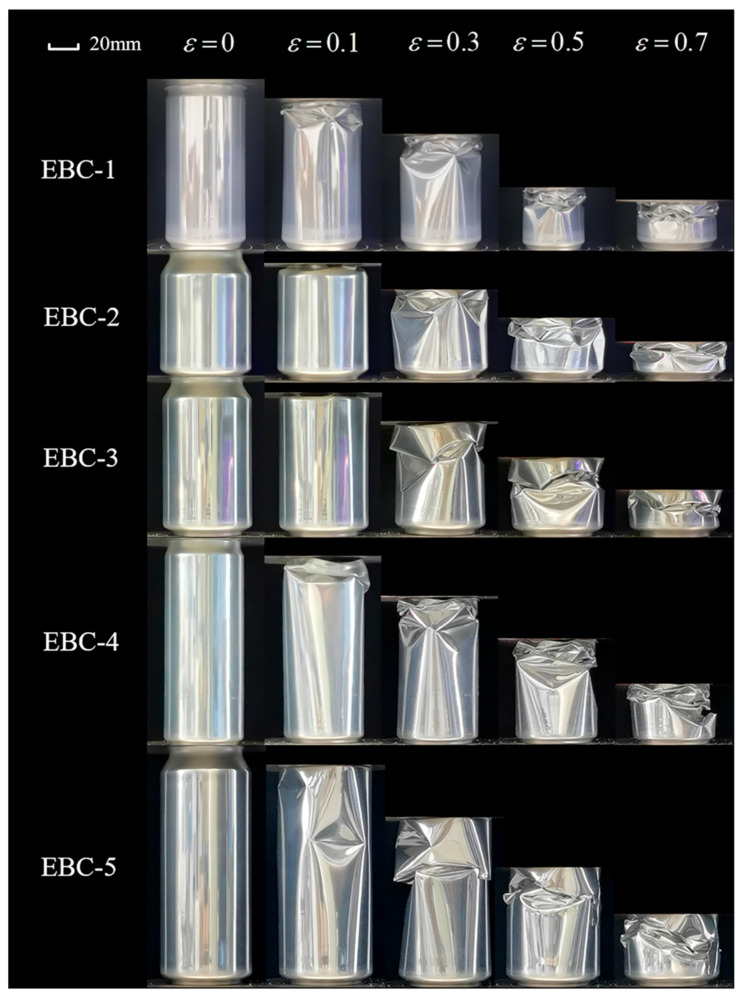
Crushing process of different types of empty beverage cans.

**Figure 8:**
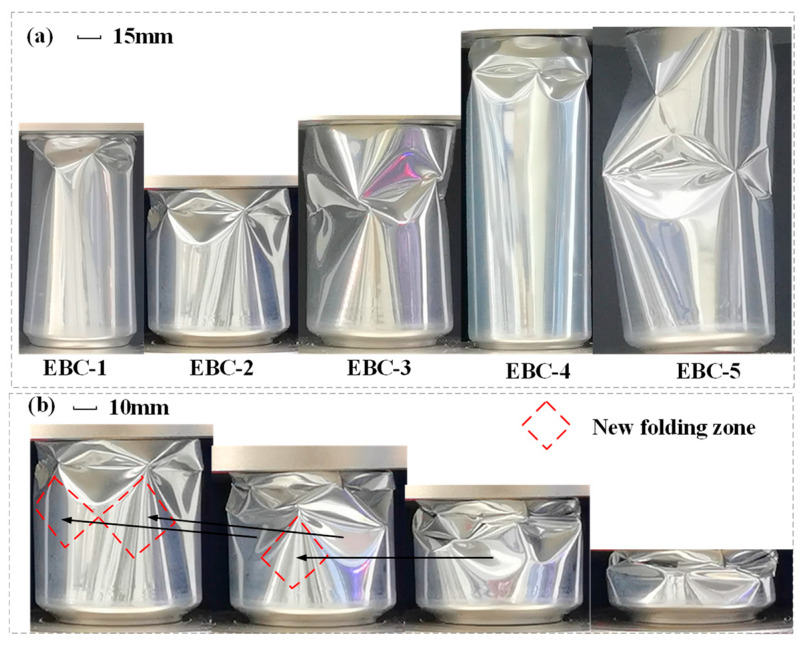
(**a**) Position of the initial crush of ECBs; (**b**) propagation of the folds of EBC-2.

As compression progresses, the pressure head makes contact with the cans body, leading to secondary buckling of the structure. The crushing process of the beverage can, depicted in Figure 7, illustrates the progression from initial buckling to collapse, reaching 70% of the total height. The illustration reveals that EBC-3 and EBC-5 cans buckle near the middle position, while EBC-1, EBC-2, and EBC-4 exhibit flexion starting from the lower part of the neck. The collapse pattern involves the formation of sharp pepper rhombus-shaped plastic hinges along the circumferential direction, which then depress inward along the folds of the horizontal diagonal lines of the rhombus. Following compaction, two adjacent rhombus-shaped hypotenuses combine to form a new rhombus-shaped plastic hinge. Local buckling occurs continuously and propagates downward until the structure is fully compacted. Figure 8a illustrates the initial buckling state of the structure, while Figure 8b demonstrates the mode of local buckling propagation in EBC-2.

**Figure 9:**
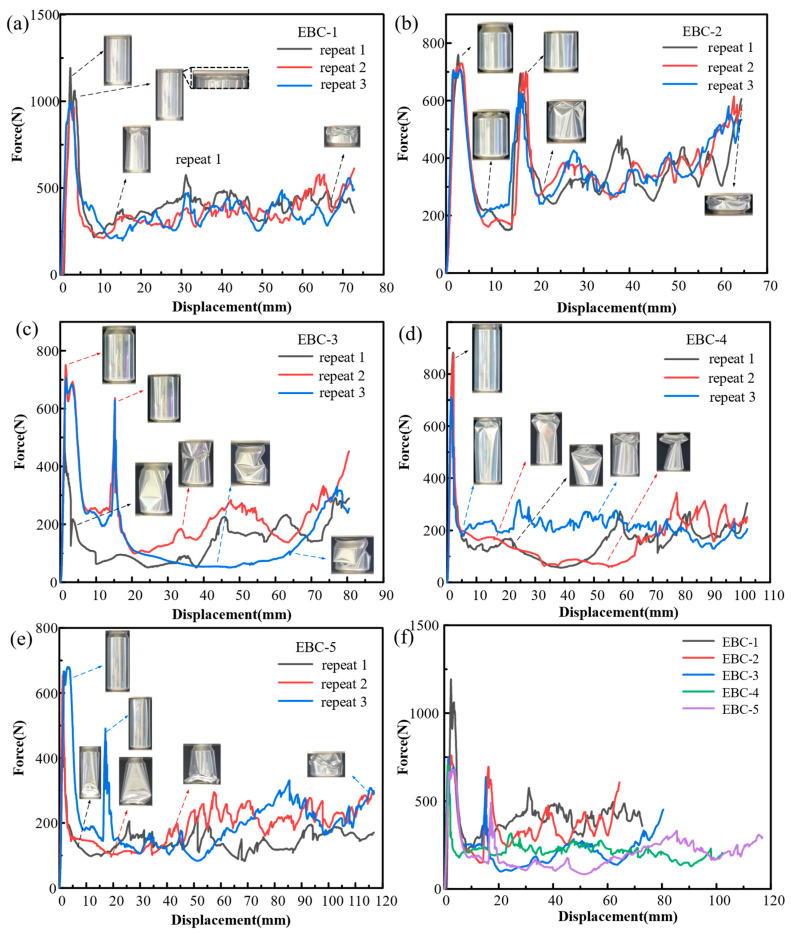
(**a**–**e**) Force-displacement curves from EBC-1 to EBC-5; (**f**) force-displacement curves for five types of EBCs.

The phenomenon of local buckling in the neck and body of the cans resulted in the appearance of two peaks in the force-displacement curve in Figure 9. Figure 9b,c,e represent the force-displacement curves of EBC-2, -3, and -5, respectively, with larger dimensions in their necks leading to more pronounced peaks in the curves. The first peak corresponds to the local buckling strength of the can neck, while the second peak corresponds to the buckling load of the can body. Figure 9a,d correspond to EBC-1 and -4 with lower neck heights, resulting in shorter intervals between the two peaks. The distance between the two peaks is determined by the height of the can neck. Figure 9f provides a summary of the force-displacement characteristics of typical samples from the five cans, revealing that EBC-1 had the highest initial peak force at 1192.9 N, while EBC-5 had the lowest initial peak force at 679.1 N. The initial peak forces are ranked in descending order as follows: EBC-1, EBC-4, EBC-2, EBC-3, and EBC-5. It is evident that necking can effectively reduce the initial peak force of the structure. Combining this with the dimensional parameters in Table 1, it can be observed that EBC-1, with the highest diameter-to-thickness ratio, has the highest platform load level, while the two groups with the smallest diameter-to-thickness ratios, EBC-3 and EBC-5, are at lower levels.

**Figure 10:**
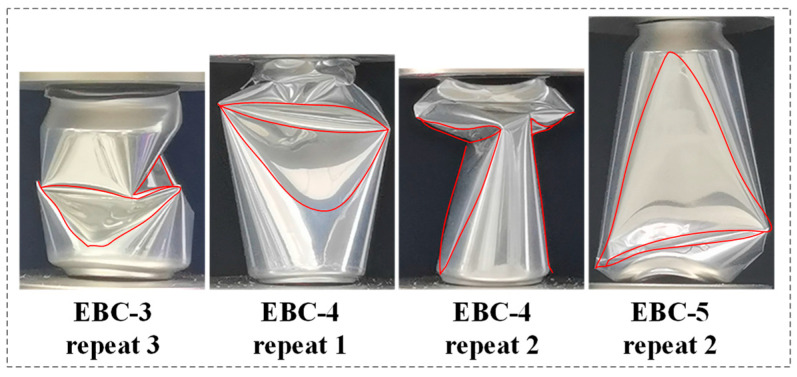
Atypical collapse patterns of empty beverage cans.

During the experiment, it was observed that EBC-3, EBC-4, and EBC-5 exhibited unusual buckling modes. The abnormal buckling modes of these beverage cans are depicted in Figure 10. The images reveal that EBC-3 and EBC-5 did not undergo local buckling in the neck initially; instead, they directly experienced significant concave buckling in the body of the can, leading to instability of the entire container. Symmetrical large concavities appeared on both sides of the middle section of the body, contributing to this instability. The force-displacement curves in Figure 9 indicate that this unconventional crushing state often accompanies low crushing forces, which can compromise the EA capacity of the structure. This phenomenon is attributed to the presence of uneven thickness and local defects during manufacturing and use. These imperfections hinder accurate predictions of collapse behavior using theoretical methods and numerical simulations. To prevent such atypical instability, the empty beverage cans were filled with polyurethane foam, enhancing the overall EA of the structure and promoting a more stable deformation mode in the filled composite structure.

**Figure 11:**
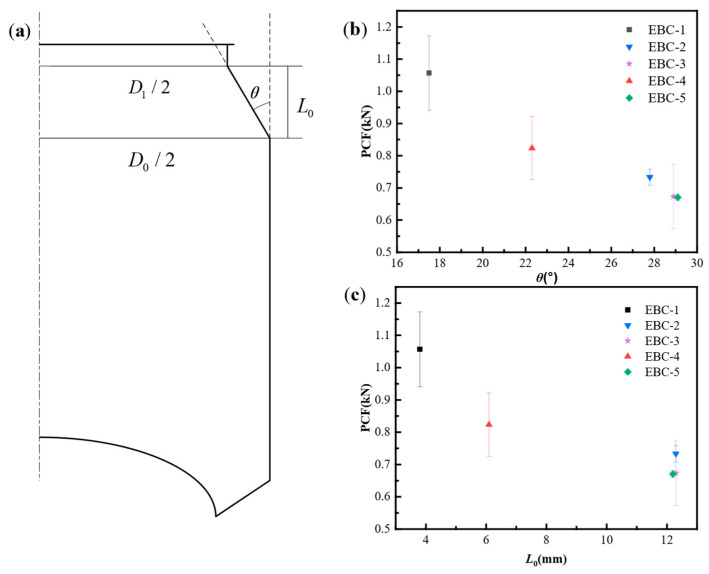
(**a**) Geometric parameters of beverage can neck; (**b**) effect of θ on the PCF of EBCs; (**c**) effect of *L*_0_ on the PCF of EBCs.

During the test on empty beverage cans, we found that under ideal conditions the initial local buckling occurs in the concave part of the neck. Therefore, it is necessary to analyze the influence of the neck size on the initial local buckling. Here we ignore the rounded corners of the structure, as well as the relationship between the bottle mouth and the bottle. The size of the lid connection is equivalent to the can neck as a frustum. Therefore, we measured the data on the neck of the beverage can according to the simplified diagram given in Figure 11a, and the measurement results are shown in Table 2.

Table 2 presents the measurements of the height (*L*_0_), maximum diameter (*D*_0_), and lowest diameter (*D*_1_) of the neck of the beverage can. By utilizing trigonometric functions, we can determine the angle between the neck of the can and the extension line of the busbar on the can body. The initial peak force results of the empty can are correlated with the angle (*θ*) and neck height (*L*_0_) as shown in Figure 11b and Figure 11c, respectively.

Figure 11b demonstrates that the initial peak force of the EBC structure is influenced by the inclination angle. As the neck inclination angle increases, the initial peak force of the EBC decreases. EBC-3 and EBC-5 have comparable dimensional parameters, resulting in similar average initial peak forces. Moving on to Figure 11c, EBC-1, with the lowest neck height, exhibits the highest initial peak force, followed by EBC-4 with the second lowest neck height. EBC-2 and EBC-3, as well as EBC-5, share similar neck heights. The initial peak force of EBC-3 closely matches that of EBC-5, while EBC-2 surpasses both due to its smaller inclination angle.

### 3.2. Crushing Behavior of Foam-Filled Cans

**Figure 12:**
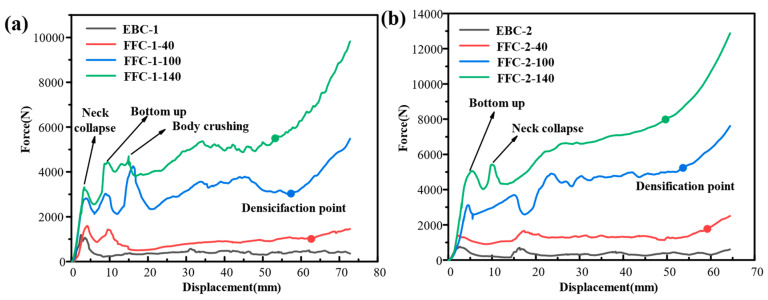
Force-displacement curves of FFCs with different densities. (**a**) FFC-1; (**b**) FFC-2.

Beverage cans were filled with three different types of polyurethane foam, with average densities of 40 kg/m^3^, 100 kg/m^3^, and 140 kg/m^3^. After allowing the foam to mature for 48 h, a quasi-static compression experiment was conducted. Force-displacement curves of the filled samples were compared with an empty can of the same type. Figure 12 illustrates the results for FFC-1 and FFC-2 beverage cans. The force-displacement curves revealed that when the can is empty or filled with 40 kg/m^3^ foam, the bottom of the can does not press into the body, resulting in a curve with two peaks. However, at densities of 100 kg/m^3^ and 140 kg/m^3^, the curves exhibited three peaks corresponding to neck crushing, the bottom pressing into the can body, and the can body buckling. Increasing the filling density transitions the crushing mode from Type II EA of empty cans to the Type I EA mode of polyurethane foam [50]; that is, the higher the density of the foam, the more obvious the enhancement in stiffness, initial peak load, and load platform.

**Figure 13:**
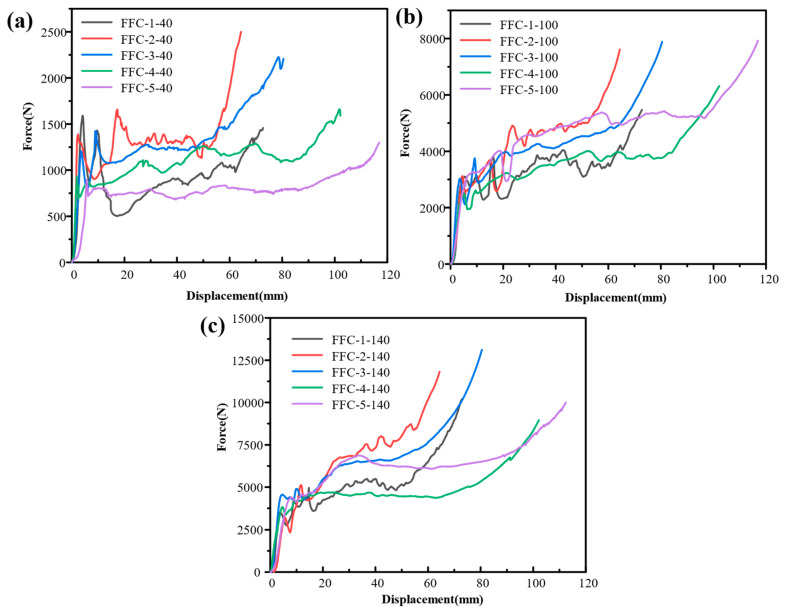
Force-displacement curves for different FFCs. (**a**) Density of 40 kg/m^3^; (**b**) density of 100 kg/m^3^; (**c**) density of 140 kg/m^3^.

Figure 13 illustrates the force-displacement curves of five beverage cans filled with polyurethane foam of varying densities. Among the cans filled with foam of the same density, the FFC-2 cans exhibit the highest platform force. Specifically, Figure 13a depicts the force-displacement curves of the cans filled with 40 kg/m^3^ polyurethane foam, showing a curve shape resembling that of a Type II energy-absorbing structure. The force level decreases rapidly after reaching peak stress, with a more horizontal compacted section compared to the cans filled with 100 kg/m^3^ and 140 kg/m^3^ foam. In Figure 13b,c, the later stage of the compaction section shows an upward slope trend in the platform section of the curve.

**Figure 14:**
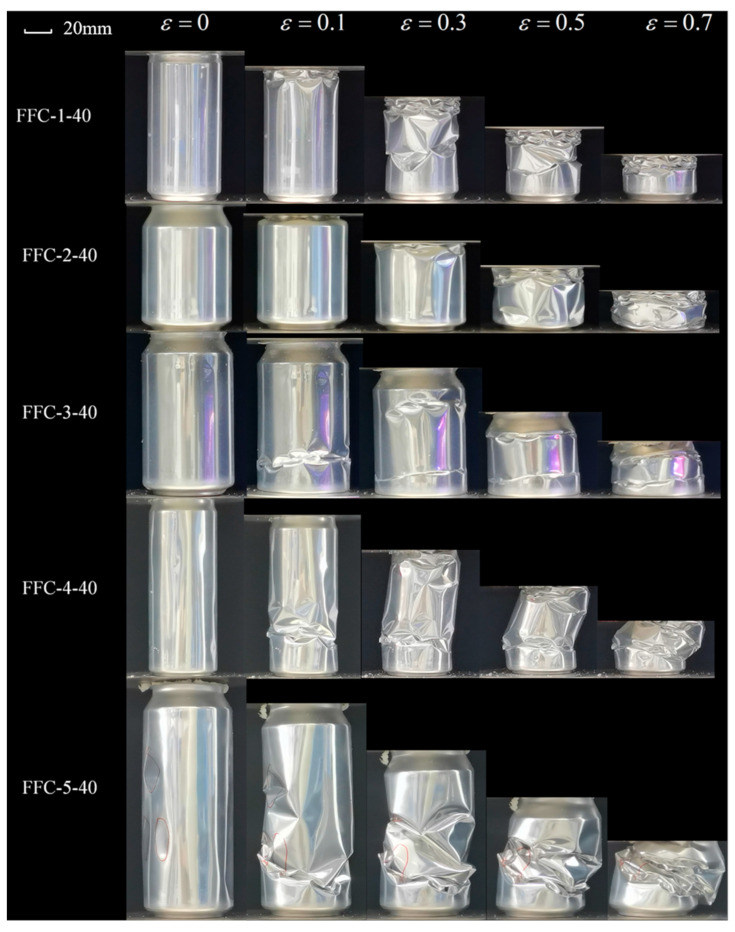
Crushing process of 40 kg/m^3^ FFCs.

**Figure 15:**
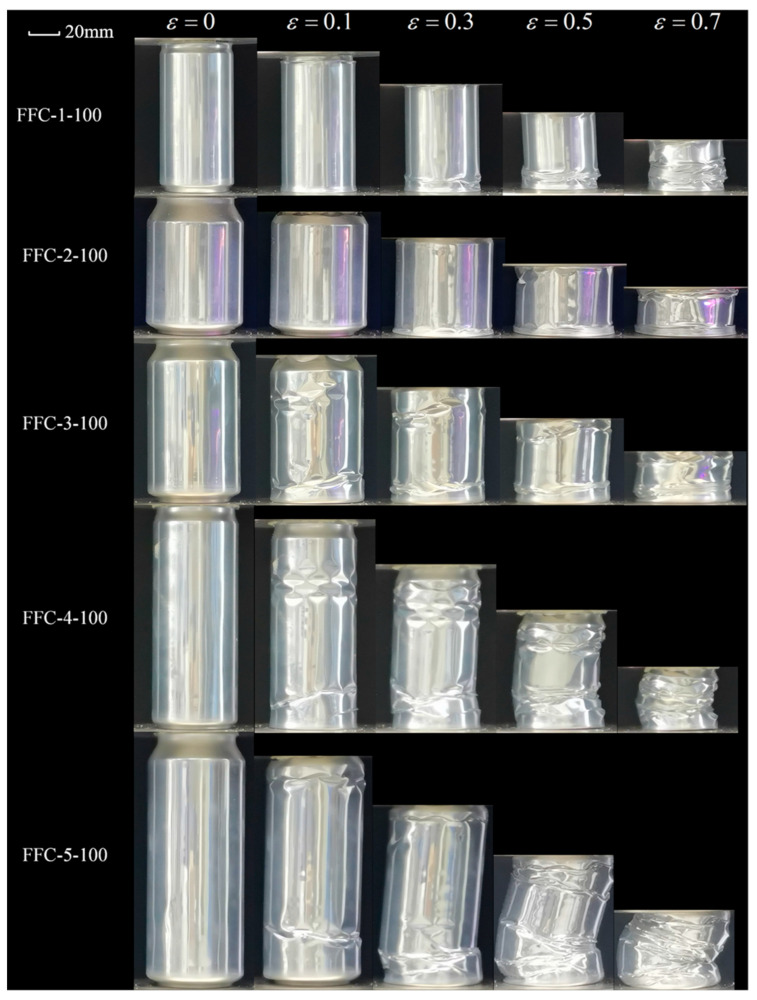
Crushing process of 100 kg/m^3^ FFCs.

**Figure 16:**
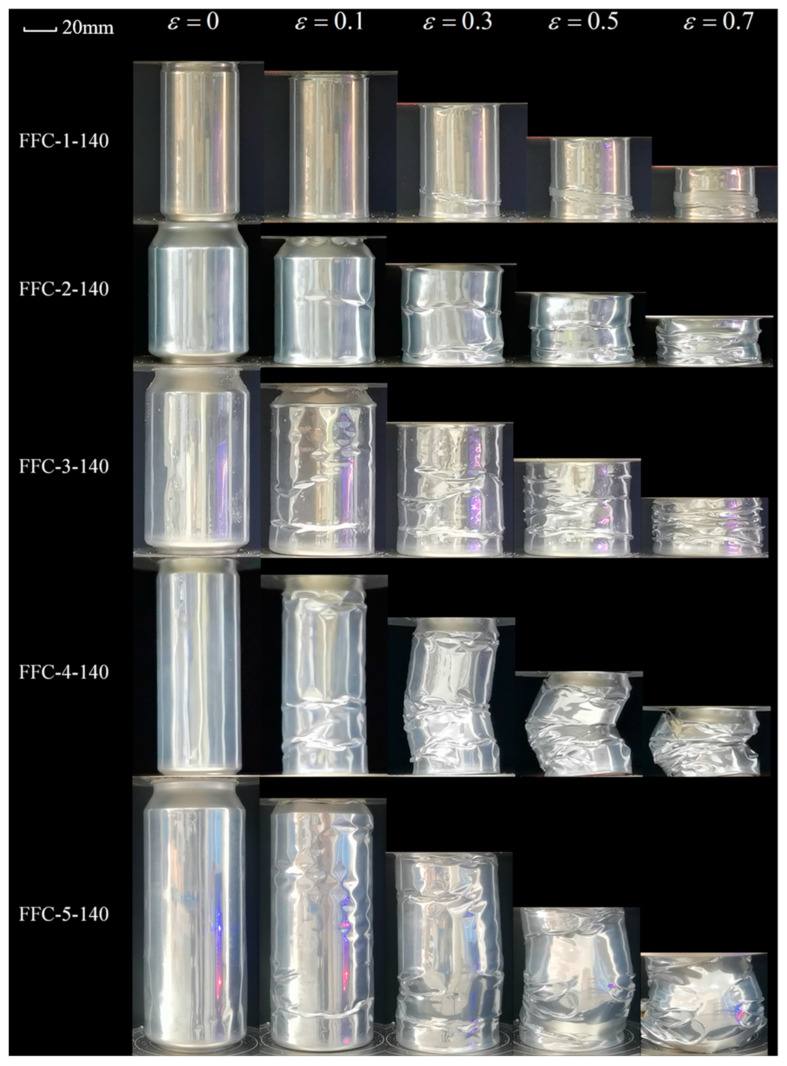
Crushing process of 140 kg/m^3^ FFCs.

Figure 14, Figure 15 and Figure 16 illustrate the crushing process of samples contained in cans filled with polyurethane foam at densities of 40 kg/m^3^, 100 kg/m^3^, and 140 kg/m^3^, respectively. The quasi-static collapse process of 40 kg/m^3^ polyurethane foam-filled cans is shown in Figure 14. For FFC-1 and FFC-2, the initial local buckling of the structure occurs at the tank neck. The tank neck is completely pressed into the inside of the can body, resulting in new inward buckling along the circumferential direction on the upper part of the can body connected to the neck. The edge of the inward buckling expands outward until it meets the edge of the adjacent concave. The entire structure then propagates axially downward in a diamond mode of progressive buckling. As this propagation occurs, new asymmetric plastic hinges emerge in the middle and lower parts of the can body, developing upward simultaneously until the structure is compacted to a predetermined height. In the case of FFC-3, the bottom is initially pressed into the can body, followed by circumferential buckling in the middle and lower parts, and axial concave buckling in the upper part as the crushing progresses. For FFC-4 and FFC-5, initial local buckling occurs in the lower part due to the structure’s height, generating large bending stress and concentrating plastic hinges on one side, resulting in a global Euler buckling mode with parallel and non-axial ends.

In Figure 15, the beverage cans were filled with polyurethane foam with a density of 100 kg/m^3^. The use of the higher density foam changed the deformation behavior of the beverage cans, resulting in the collapse of the top and bottom of all five types of beverage cans and their eventual compression into the interior of the cans. Symmetric folds are observed in the bodies of the FFC-1 and FFC-2, indicating a change from an asymmetric diamond mode to a symmetric concertina mode. Symmetric folds are observed in the upper portion of the bodies of the FFC-3, FFC-4, and FFC-5. Plastic hinges gradually appear on the upper and lower parts of the cans. It is noteworthy that FFC-3 and FFC-4 have multiple sets of circumferential diamond-shaped indented plastic hinges on the upper part of the can. These smaller and more numerous diamond-shaped folds require more energy for compression than the empty and 40 kg/m^3^ filled cans. The largest can, FFC-5, exhibited cylindrical bending during compression with the folds concentrated on one side of the tube.

As the filling density increased to 140 kg/m^3^, Figure 16 shows the bending instability of FFC-4 and FFC-5. The maximum deflection position is in the middle of the can body, with folds concentrated on one side. The curve in Figure 13c displayed a relatively stable crush curve, albeit at a lower level compared to other samples. This indicates that the EA capacity of the structure in its bending state is lower than in axial crushing conditions. Among the other three groups of specimens, FFC-1 exhibits symmetrical concertina mode compression, while FFC-2 and FFC-3 displays pairs of circumferential folds simultaneously with compression, maintaining a high load level. The results suggest that with increased filling density, the EA of the beverage can is influenced by internal filling.

**Figure 17:**
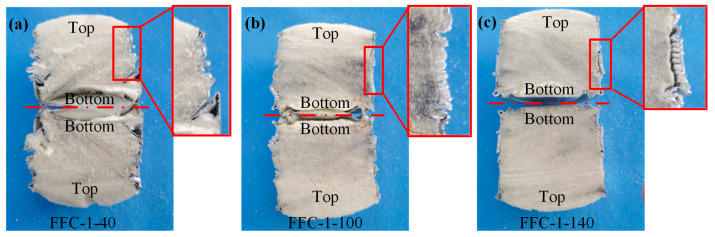
Sections of foam-filled cans with different densities. (**a**) FFC-1-40; (**b**) FFC-1-100; (**c**) FFC-1-140.

Figure 17 illustrates the distribution of plastic hinge lines for three groups of FFC-1-40, 100, and 140 beverage cans post cutting. Notably, in the EBC-1, all tube walls exhibit progressive buckling. With increasing foam density, the deformation mode of the can body transitions from a diamond mode to a mixed mode and ultimately to a concertina mode. At densities of 40 kg/m^3^ and 100 kg/m^3^, the tank body displays wrinkles resulting from continuous progressive buckling that press against the foam. However, at a density of 140 kg/m^3^, some regular and fine wrinkles detach from the internal foam.

**Figure 18:**
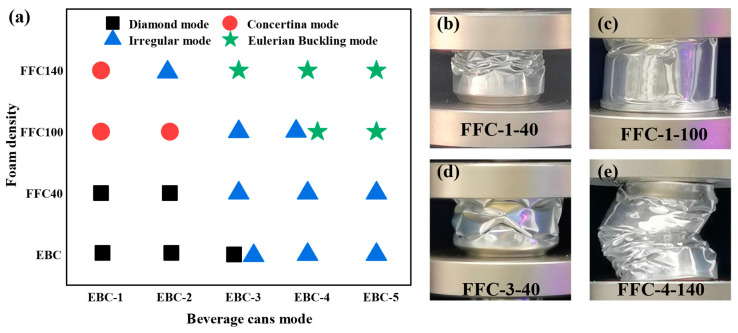
(**a**) Summary of deformation patterns of EBCs and FFCs; (**b**) diamond mode; (**c**) concertina mode; (**d**) irregular mode; (**e**) Euler buckling mode.

The deformation modes of all specimens were summarized and divided into four categories: (1) diamond mode, (2) concertina mode, (3) irregular mode, and (4) global bending mode. Figure 18b–e illustrate typical patterns for each category. The results indicate that with increasing foam density, more folds appear on the can body, both along the generatrix direction of the cylinder and along the circumferential direction. Additionally, the higher foam density alters the folding behavior of the cans, transitioning smaller sizes (EBC-1, EBC-2, EBC-3) from a diamond pattern to an accordion pattern, while larger sizes (EBC-4, EBC-5) gradually shift from irregular deformation to a global bending mode. The deformation patterns for all cases are documented in Figure 18a.

### 3.3. Energy Absorption of Beverage Cans

#### 3.3.1. Crashworthiness Indicators

The article uses several widely used EA evaluation indicators to evaluate the EA capacity of cans. They are energy absorption (EA), specific energy absorption (SEA), peak crushing force (PCF), mean crushing force (MCF), and crush force efficiency (CFE), which are obtained directly or indirectly from the force-displacement curves obtained above.

The introduction of polyurethane foam resulted in premature compaction of the structure. Different structures had varying compaction points. Setting uniform compaction points artificially can lead to inaccuracies. Hence, to prevent manual setting of compaction points and mitigate interference with crashworthiness parameters, the concept of effective compression stroke *h*_0_ was introduced. This parameter signifies the compression stroke from the initial compression of the structure to its compaction point. Details on calculating the compaction point can be found in [51].

In order to objectively define the location of the compaction point of the structure, energy absorption efficiency (EAE) is defined as

(2) $$f=\frac{\int_{0}^{h}F(x)dx}{F_{\max}}$$

where h represents the compression stroke and $F_{\max}$ is the maximum force within the interval [0, *h*], excluding the PCF. With the increase in the compression stroke, there exists a peak value for the EAE, and the corresponding compression stroke at this peak value is denoted as the effective compression stroke $h_{0}$. The introduction of the effective compression stroke allows for a more accurate and objective assessment of the optimal compression stroke for the structure, while also mitigating the impact of artificially set compaction points on test results.

Crashworthiness parameters were calculated based on the force-displacement curves within the effective compression stroke *h*_0_. EA is determined as the integral of the load-displacement curve in the displacement direction, denoted as

(3) $$EA=\int_{0}^{h_{0}}F(x)dx$$

where $h_{0}$ represents the effective compression stroke and F(x) is the corresponding load-displacement curve. EA quantifies the capacity of energy dissipation systems.

Mass is the metric we are concerned with in the design of energy-absorbing structures, thus we introduced the concept of specific energy absorption (SEA), which reflects the EA capacity of a unit mass of the structure. Its expression is

(4) $$SEA=\frac{EA}{m}=\frac{\int_{0}^{h}F(x)dx}{m}$$

where *m* is the mass of the energy-absorbing structure.

PCF refers to the initial peak force in the load curve, which can be directly read from the load-displacement curve. Excessive PCF in energy-absorbing structures can lead to harm to the occupants or equipment being protected.

MCF is defined as the average crushing force experienced by the structure during the effective compression stroke. Its expression is

(5) $$MCF=\frac{\int_{0}^{h_{0}}F(x)dx}{h_{0}}$$

MCF corresponds to the average level of crushing force during the compression stroke.

PCF and MCF are important parameters to measure the magnitude of deceleration suffered by the occupant. In order to quantify the relationship between PCF and MCF, CFE is defined as the ratio of the average force to the initial peak force. It is calculated using the following formula,

(6) $$CFE=\frac{MCF}{PCF}$$

**Figure 19:**
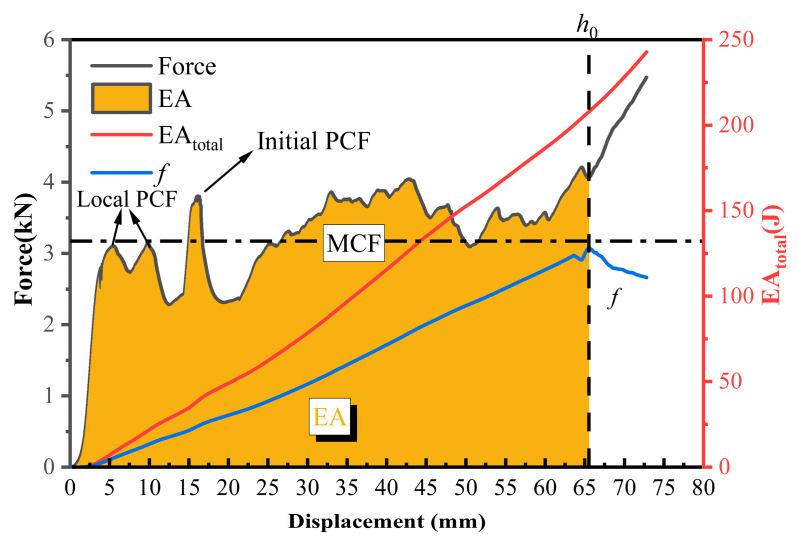
Force-displacement curve and EA curve for typical specimen FFC-1-100.

Figure 19 shows the force-displacement curve of a sample. Each parameter is identified in the figure according to the definition above. The densification point is denoted by *h*_0_, the area under the force-displacement curve from 0 to *h*_0_ is represented by EA, and PCF signifies the compression process. The maximum peak force in MCF corresponds to the average load within the range from 0 to *h*_0_.

#### 3.3.2. Energy Absorption in Axial Compression

**Figure 20:**
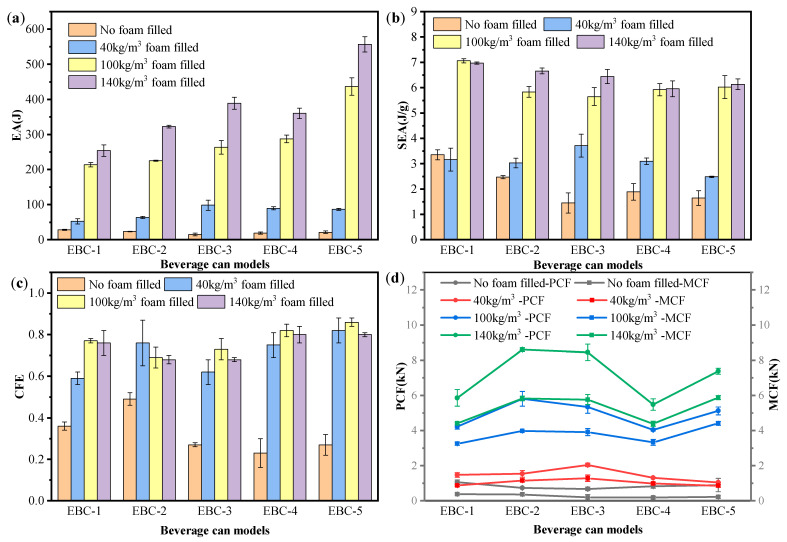
Comparison of results for crashworthiness parameters. (**a**) EA; (**b**) SEA; (**c**) CFE; (**d**) PCF and MCF.

The crashworthiness parameters of each specimen are summarized in Figure 20. Figure 20a presents the EA of various types of beverage cans under unfilled and different density foam filling conditions. It can be observed that foam filling effectively increases the EA of the composite structure. By comparison, FFC-1-40, FFC-1-100, and FFC-1-140 show EA enhancements of 89.3%, 668.6%, and 813.8% respectively, when compared to the EBC-1. Similarly, for EBC-2, the EA increments are 174.6%, 869.6%, and 1287.6%; for EBC-3, they are 579.5%, 1712.5%, and 2580.8%; for EBC-4, they are 383.5%, 1450.5%, and 1843.6%; and for EBC-5, they are 307.8%, 1949.3%, and 2515.4%, respectively. With an increase in foam filling density, the EA capacity of the structure significantly increases. The EBC-3 and EBC-5 filled with 140 kg/m^3^ foam exhibit the highest increase in EA, reaching increments of 2580.8% and 2515.4%, respectively.

Figure 20b illustrates the SEA parameters of EBCs and FFCs with varying densities. The SEA of EBC-1 is 3.35 J/g, the highest among all the empty cans. When filled with 40 kg/m^3^ foam, the structure’s SEA is reduced by 5.7% compared to EBC-1. As the foam density increases to 100 kg/m^3^, the specific energy absorption increases by 110.7% compared to EBC-1. Further, at a foam density of 140 kg/m^3^, the SEA increases by 108.1% compared to EBC-1, showing a slight decrease from the 100 kg/m^3^ foam filling scenario. The SEA of EBC-2 is 2.47 J/g. In comparison to EBC-2, the foam filling densities are 40 kg/m^3^, 100 kg/m^3^, and 140 kg/m^3^. The specific absorption energy can be increased by 22.7%, 136%, and 169.6%, respectively, with these foam densities. The SEA of EBC-3 is 1.45 J/g, and the improvements brought by foam filling are 155.9%, 289.7%, and 344.1%, respectively. The SEA of EBC-4 is 1.89 J/g. Compared with the empty can, the improvements brought by foam filling are 64%, 213.2%, and 215.3%, respectively. The SEA of EBC-5 is 1.65 J/g, and the improvements brought by foam filling are 50.3%, 264.8%, and 271.5%, respectively. For EBC-4 and EBC-5, filling with 140 kg/m^3^ foam leads to a limited increase in SEA compared to filling with 100 kg/m^3^ foam. These findings suggest that higher foam density can enhance the SEA of the composite structure, but there exists an optimal foam density that maximizes this parameter. For instance, FFC-1-100 achieves a maximum SEA value of 7.06 J/g.

The CFE parameter is determined by both PCF and MCF. From Figure 18c, it is observed that the CFE of foam-filled beverage cans is greater than that of empty cans of the same type, suggesting that foam filling is advantageous for enhancing the CFE of composite structures. However, it can be seen that for all types of EBCs, the CFE values of FFCs with 140 kg/m^3^ foam are lower than those of FFC-100 of the same type. For EBC-1, -3, -4, and -5, FFCs with 100 kg/m^3^ foam exhibit the highest CFE, while for EBC-2, FFCs with 40 kg/m^3^ foam have the highest CFE value. From Figure 20d, it can be observed that for EBC-2, as the density increases, the initial peak stress significantly increases. Therefore, it can be inferred that the decrease in CFE value for FFC-2 is due to the increase in structural stiffness after foam filling, leading to a greater increase in initial peak force compared to the increase in MCF. Thus, in this experiment, CFE does not increase continuously with an increase in foam density.

### 3.4. Interaction Effects of Foam Filling

**Figure 21:**
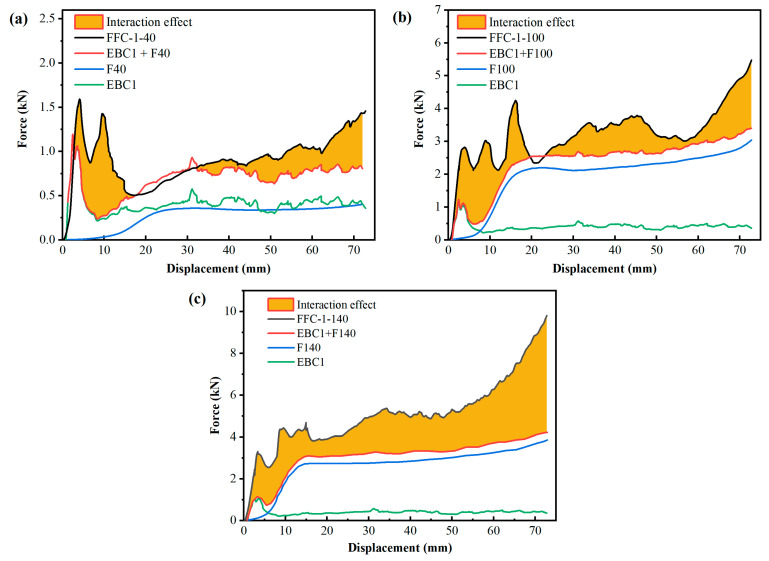
Interaction effect of various densities of foam filled in EBC-1. (**a**) 40 kg/m^3^; (**b**) 100 kg/m^3^; (**c**) 140 kg/m^3^, where F40, F100 and F140 are the density of the polyurethane foam, and EBC1+F40 is the sum of the EBC1 curve plus the F40 curve.

To quantify the interaction effect of foam filling on the EA of beverage cans, the separate calculations for each type of beverage can and corresponding foam with varying densities were conducted. The total EA of the empty can and foam was then compared with the EA value of the filled can. The EA of the foam was determined by integrating the force-displacement curves outlined in Section 2.3. Using EBC-1 beverage cans as a reference, Figure 21 displays force-displacement curves for foam filling with three varying densities. Analysis of the curve reveals that the EA capacity of the filled can surpasses the combined EA values of the empty can and the respective foam. This suggests a synergistic effect between the foam and the empty beverage can under compression. To investigate the relationship between EA in empty beverage cans, foam, and their interaction effects, we calculated the energy absorption ratio for five beverage cans filled with foam of different densities. The EA of empty beverage cans was determined by averaging the results of tests on unfilled cans, while the EA of foam was calculated using the densification strain measured in tests on filled cans. The interaction effect was quantified as the difference between the EA of the filled can, the EA of the empty beverage can, and the EA of the foam.

**Figure 22:**
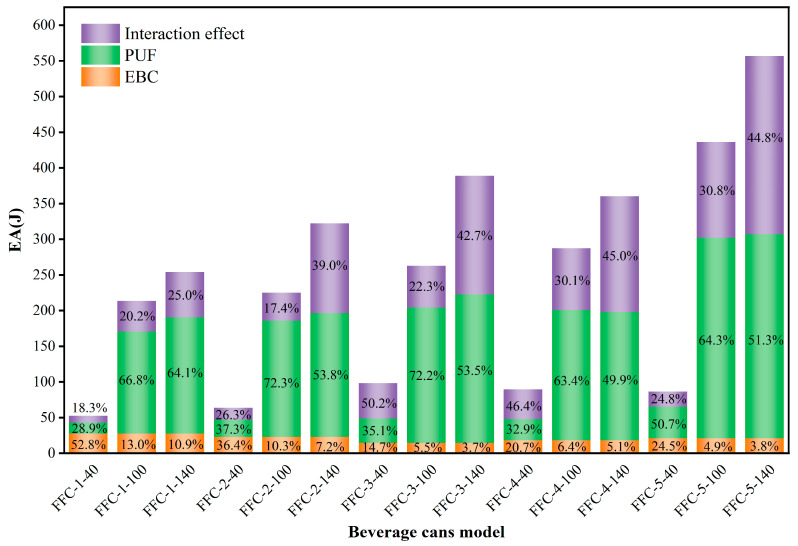
Beverage cans examined in this study demonstrate superior SEA when compared to similar structures.

The results of the calculations are shown in Figure 22, which clearly illustrates that as the foam density increases, the proportion of EA attributed to the interactive part also increases. Analysis of the test results indicates that the reason for the increase in interaction effects is multifaceted. The addition of foam enhances the overall stiffness of the composite structure, thereby altering the deformation mode of the beverage can. This results in more folds in the can body, leading to higher energy consumption. In addition, lower density foam can introduce defects such as voids and uneven density during the foaming process. In addition, after 40 kg/m^3^ foam is added to the can, it may shrink over time, causing the inner wall of the can to become concave, resulting in macroscopic defects. However, this problem does not occur with 100 kg/m^3^ and 140 kg/m^3^ foam, highlighting that higher-density foam filling increases the interaction effect between the foam and the can.

**Figure 23:**
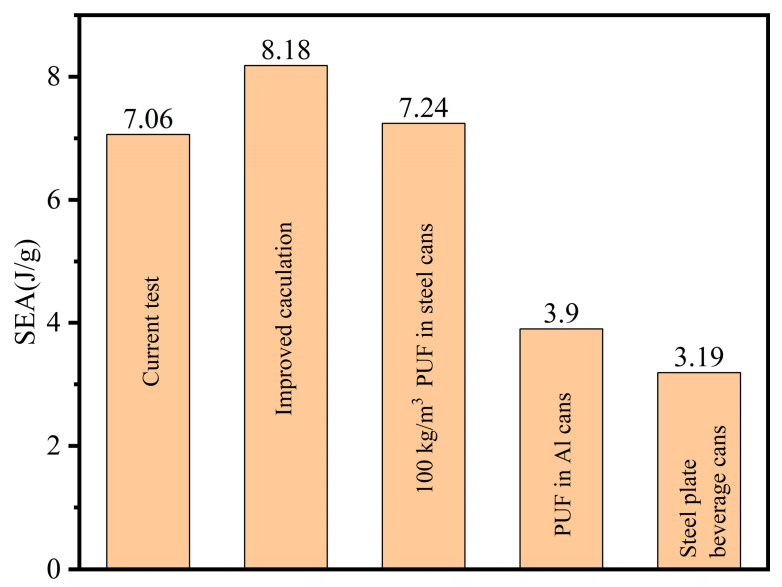
Comparison of SEA of similarly structures, where Al stands for aluminum [46,47,52].

As illustrated in Figure 23, at a consistent density of 100 kg/m^3^, the SEA of EBC-1 aluminum beverage cans matches that of steel beverage cans discussed in [47]. Notably, the SEA calculation in this study is based on the EA calculation prior to the compaction point. Following the methodology outlined in [47], the SEA of EBC-1 throughout the entire stroke was calculated to be 8.18 J/g, surpassing that of steel beverage cans. Furthermore, the SEA data of EBC-1 outperform the data presented in [46,52].

As the global security landscape grows increasingly precarious, new terrorist attack methods pose fresh threats to buildings and vehicles. The low-cost recyclable energy absorber outlined in this study offers a versatile solution for mitigating damage from collisions, impacts, and explosions. By serving as a sacrificial layer atop structures, this absorber effectively shields against explosives dropped by low-altitude drones, while also providing additional insulation for the buildings. The key benefits of this technology include easy access to raw materials, a straightforward production process, rapid scalability, and simplified deployment. To further maximize its utility, we introduce the concept of combined application with support materials (Figures S1–S3). This approach not only expands the range of available raw materials but also enhances structural stability. By manipulating the arrangement of these absorbers, various energy absorption responses can be achieved, paving the way for passive adaptive energy absorption research.

## 4. Conclusions

The purpose of this study is to suggest a cost-effective, recyclable, and eco-friendly composite energy-absorbing structure. Waste beverage cans were utilized and filled with polyurethane foam to act as energy absorbers. The research included a systematic axial compression test on composite energy-absorbing structures of various sizes of cans and filling densities of foam. The findings of the study indicate:

(1). The EA modes of all five types of empty beverage cans are classified as Type II. The primary failure mode is local buckling, with the location of buckling being influenced by the size parameters of the cans. The deformation modes of empty beverage cans have important effects on the manufacturing and use process. The EA performance of beverage cans with local defects will be significantly reduced.
(2). Filling cans with polyurethane foam changes the deformation mode of a structure. As the foam density increases, the deformation mode of smaller-sized beverage cans changes from diamond mode to concertina mode, while larger-sized beverage cans are gradually deformed by the polyurethane foam. The deformation mode approaches Euler bending.
(3). Compared to empty beverage cans, adding polyurethane foam shifts the EA from solely Type II to a combination of Type I and II, enhancing overall crashworthiness metrics such as EA, SEA, PCF, MCF, and CFE. Across all five can types, EA rises with higher foam density, and SEA generally trends upward as well.
(4). There is a coupled energy-absorbing phenomenon between the foam filling and the can body, as evidenced by the increased EA capacity of foam-filled cans compared to the cumulative EA of the individual components. As the filling density increases from 100 kg/m^3^ to 140 kg/m^3^, there is a corresponding increase in the proportion of energy absorbed by the interaction effects.
(5). FFC-5-140 exhibits the highest EA among the five types of FFCs, whereas FFC-1-100 demonstrates the best SEA. The larger EBC-5 can is capable of absorbing more energy, while the EBC-1 can, with a smaller aspect ratio, shows advantages in SEA when filled with polyurethane compared to other structures.

This research offers a foundational basis for the practical use of discarded beverage cans in energy-absorbing structures, promoting sustainable economic growth and environmental conservation. However, some aspects have not been fully reported due to the limited research period, such as corresponding simulations, higher loading rates, impact crush behavior, and other types of foam filling, which shall be addressed in future work.

## Figures and Tables

**Table 1:** Geometric parameters of beverage cans.

Specimen	Capacity (mL)	*D*_0_ (mm)	*T* (mm)	*H*_0_ (mm)	*H*_1_ (mm)
EBC-1	190	53	0.153	104	93
EBC-2	250	66	0.134	92	69
EBC-3	330	66	0.112	115	93
EBC-4	330	57	0.118	146	130
EBC-5	500	66	0.116	167	143

**Table 2:** Dimensional parameters of can necks.

Specimen	*L*_0_ (mm)	*D*_1_ (mm)	*D*_0_ (mm)	θ (°)
EBC-1	3.8	50.6	53.0	17.5
EBC-2	12.3	53.0	66.0	27.8
EBC-3	12.3	52.4	66.0	28.9
EBC-4	6.1	52.0	57.0	22.3
EBC-5	12.2	52.4	66.0	29.1

## Data Availability

Data will be made available on request.

## Supplementary Materials

The following supporting information can be downloaded at: https://www.mdpi.com/article/10.3390/ma17112655/s1, Figure S1: The style of combined configuration, Figure S2: Crushing of different structures, Figure S3: Energy absorption in combined and original structures. (a) Force-displacement curves; (b) EA and SEA; Table S1: Thickness of beverage can bodies, Table S2: Test results of all specimens.
