# Supplementary material for: On the Crush Behavior and Energy Absorption of Sustainable Beverage Cans and Their Polyurethane Foam-Filled Structures: An Experimental Study
Source: Materials (Basel). 2024 May 31;17(11):2655. doi: 10.3390/ma17112655 (PMC11174009; doi:10.3390/ma17112655)
Supplement: Supplementary file 1 [file materials-17-02655-s001.zip › materials-2950821-supplementary.pdf]

On the Crush Behavior and Energy Absorption of Sustainable Beverage Cans and their Polyurethane Foam-Filled Structures: An Experimental Study

Supplementary materials

Table S1 provides further details on our measurement process, which utilized a micrometer with 0.01 accuracy. Measurements were taken at the upper, middle, and lower sections of the can body, with the data sets labeled as T1, T2, and T3, respectively.

Table S1 Thickness of beverage can bodies

TYPE	T_1(mm)	T_2(mm)	T_3(mm)	T_{avg}(mm)	SD
EBC-1	0.145	0.160	0.154	0.153	0.00755
EBC-2	0.140	0.128	0.134	0.134	0.006
EBC-3	0.107	0.121	0.108	0.112	0.00781
EBC-4	0.115	0.116	0.123	0.118	0.00436
EBC-5	0.105	0.115	0.128	0.116	0.01153

We conducted 60 compression tests for 5 different beverage cans and 3 different foam densities, including empty beverage cans. The test results are given in the Table S2

Table S2 Test results of all specimens

Specimen	EA	SEA	PCF	MCF	CFE	Crush mode
EBC-1-1	29.70	3.58	1.19	0.10	0.34	Diamond mode
EBC-1-2	27.07	3.26	0.99	0.37	0.37	Diamond mode
EBC-1-3	26.67	3.21	0.99	0.37	0.37	Diamond mode
average	27.81	3.35	1.06	0.38	0.36	
SD	1.65	0.20	0.12	0.02	0.02	
EBC-2-1	22.54	2.40	0.76	0.35	0.46	Diamond mode
EBC-2-2	23.48	2.50	0.73	0.36	0.50	Diamond mode
EBC-2-3	23.63	2.51	0.71	0.37	0.52	Diamond mode
average	23.22	2.47	0.73	0.36	0.49	
SD	0.59	0.06	0.03	0.01	0.03	
EBC-3-1	18.98	1.90	0.75	0.24	0.31	Diamond mode
EBC-3-2	11.54	1.15	0.56	0.14	0.26	Irregular mode
EBC-3-3	13.00	1.30	0.71	0.16	0.23	Irregular mode
average	14.51	1.45	0.67	0.18	0.27	
SD	3.94	0.40	0.10	0.05	0.01	
EBC-4-1	16.46	1.68	0.88	0.16	0.18	Irregular mode

EBC-4-2	16.95	1.73	0.88	0.17	0.19	Irregular mode
EBC-4-3	22.21	2.27	0.71	0.22	0.31	Irregular mode
average	18.54	1.89	0.82	0.18	0.23	
SD	3.19	0.33	0.10	0.03	0.07	
EBC-5-1	17.10	1.33	1.33	0.22	0.22	Irregular mode
EBC-5-2	22.72	1.76	0.66	0.19	0.30	Irregular mode
EBC-5-3	24.04	1.86	0.68	0.21	0.30	Irregular mode
average	21.29	1.65	0.89	0.21	0.27	
SD	3.68	0.29	0.38	0.01	0.05	
FFC-1-40-1	60.95	3.67	1.59	0.88	0.55	Diamond mode
FFC-1-40-2	46.58	2.81	1.34	0.81	0.61	Diamond mode
FFC-1-40-3	50.43	3.00	1.49	0.91	0.61	Diamond mode
average	52.65	3.16	1.47	0.87	0.59	
SD	7.44	0.45	0.13	0.05	0.03	
FFC-2-40-1	66.90	3.20	1.66	1.23	0.74	Diamond mode
FFC-2-40-2	63.84	3.07	1.31	1.14	0.87	Diamond mode
FFC-2-40-3	60.53	2.83	1.63	1.08	0.66	Diamond mode
average	63.76	3.03	1.53	1.15	0.76	
SD	3.19	0.19	0.19	0.08	0.11	
FFC-3-40-1	106.10	3.90	2.10	1.34	0.64	Irregular mode
FFC-3-40-2	81.98	3.20	1.92	1.07	0.56	Irregular mode
FFC-3-40-3	107.38	4.04	2.10	1.42	0.67	Irregular mode
average	98.59	3.71	2.04	1.28	0.62	
SD	14.39	0.45	0.10	0.18	0.06	
FFC-4-40-1	94.11	3.47	1.29	1.06	0.82	Irregular mode
FFC-4-40-2	85.06	3.26	1.30	0.95	0.73	Irregular mode
FFC-4-40-3	89.77	3.49	1.33	0.94	0.71	Irregular mode
average	89.65	3.10	1.31	0.98	0.75	
SD	4.53	0.13	0.02	0.07	0.06	
FFC-5-40-1	84.48	2.45	1.03	0.77	0.75	Irregular mode
FFC-5-40-2	89.96	2.51	1.05	0.89	0.85	Irregular mode
FFC-5-40-3	86.02	2.47	1.04	0.88	0.85	Irregular mode
average	86.82	2.48	1.04	0.85	0.82	
SD	2.83	0.03	0.01	0.07	0.06	
FFC-1-100-1	207.30	6.98	4.06	3.17	0.78	Concertina mode
FFC-1-100-2	219.87	7.14	4.34	3.34	0.77	Concertina mode
FFC-1-100-3	214.12	7.07	4.27	3.23	0.76	Concertina mode
average	213.76	7.06	4.22	3.25	0.77	
SD	6.29	0.08	0.14	0.09	0.01	

FFC-2-100-1	223.14	5.78	5.49	3.99	0.73	Concertina mode
FFC-2-100-2	226.21	5.66	6.28	4.00	0.64	Concertina mode
FFC-2-100-3	226.04	6.06	5.66	3.93	0.69	Concertina mode
average	225.13	5.83	5.81	3.97	0.69	
SD	1.73	0.21	0.42	0.04	0.05	
FFC-3-100-1	248.66	5.45	4.96	3.87	0.78	Irregular mode
FFC-3-100-2	284.99	6.05	5.68	4.12	0.73	Irregular mode
FFC-3-100-3	255.35	5.44	5.42	3.73	0.69	Irregular mode
average	263.00	5.65	5.35	3.91	0.73	
SD	19.33	0.35	0.37	0.20	0.05	
FFC-4-100-1	280.75	5.83	4.01	3.33	0.83	Euler buckling mode
FFC-4-100-2	281.64	5.74	3.99	3.14	0.79	Euler buckling mode
FFC-4-100-3	300.01	6.20	4.12	3.48	0.85	Irregular mode
average	287.47	5.92	4.04	3.32	0.82	
SD	10.87	0.24	0.07	0.17	0.03	
FFC-5-100-1	437.35	6.04	5.38	4.53	0.84	Euler buckling mode
FFC-5-100-2	460.44	6.47	4.99	4.38	0.88	Euler buckling mode
FFC-5-100-3	411.13	5.56	5.00	4.31	0.86	Euler buckling mode
average	436.31	6.02	5.12	4.41	0.86	
SD	24.67	0.45	0.22	0.11	0.02	
FFC-1-140-1	261.20	7.02	5.99	4.45	0.74	Concertina mode
FFC-1-140-2	235.43	6.94	5.34	4.40	0.82	Concertina mode
FFC-1-140-3	265.73	6.96	6.25	4.37	0.70	Concertina mode
average	254.12	6.97	5.86	4.41	0.76	
SD	16.34	0.04	0.47	0.04	0.06	
FFC-2-140-1	318.32	6.58	8.62	5.95	0.69	Irregular mode
FFC-2-140-2	322.77	6.78	8.69	5.70	0.66	Irregular mode
FFC-2-140-3	325.48	6.62	8.48	5.85	0.69	Irregular mode
average	322.19	6.66	8.60	5.83	0.68	Irregular mode
SD	3.61	0.11	0.11	0.13	0.02	
FFC-3-140-1	393.81	6.55	8.58	5.97	0.70	Euler buckling mode
FFC-3-140-2	403.09	6.64	8.84	5.89	0.67	Euler buckling mode
FFC-3-140-3	370.06	6.12	7.93	5.45	0.69	Euler buckling mode
average	388.99	6.44	8.45	5.77	0.68	
SD	17.03	0.28	0.47	0.28	0.01	
FFC-4-140-1	371.19	6.22	5.83	4.54	0.78	Euler buckling mode
FFC-4-140-2	366.57	6.03	5.42	4.24	0.78	Euler buckling mode
FFC-4-140-3	343.28	5.62	5.18	4.37	0.84	Euler buckling mode
average	360.35	5.96	5.48	4.38	0.80	

SD	14.96	0.31	0.33	0.15	0.04	
FFC-5-140-1	567.98	5.99	7.48	5.85	0.78	Euler buckling mode
FFC-5-140-2	570.53	6.37	7.48	6.00	0.80	Euler buckling mode
FFC-5-140-3	531.96	6.03	7.19	5.78	0.80	Euler buckling mode
average	556.82	6.13	7.38	5.88	0.80	
SD	21.57	0.21	0.17	0.11	0.01	

Inspired by passive adaptive energy-absorbing structures, we propose combining discarded beverage cans to enhance material utilization. This approach aims to utilize a wider range of solid waste beverage cans and offer increased design flexibility. By adjusting the combined structure and filling density, engineering designers can achieve greater design freedom, enabling adaptation to various energy absorption scenarios while enhancing structural strength and energy absorption capacity.

In this study, the nesting process for beverage cans involved placing a smaller diameter can inside a larger diameter can and filling the space between them with foam. This sandwich structure enhances the axial and lateral pressure resistance of the cans. Figure S1 illustrates various nesting solutions, with the possibility of combining multiple solutions based on the size of the beverage cans.

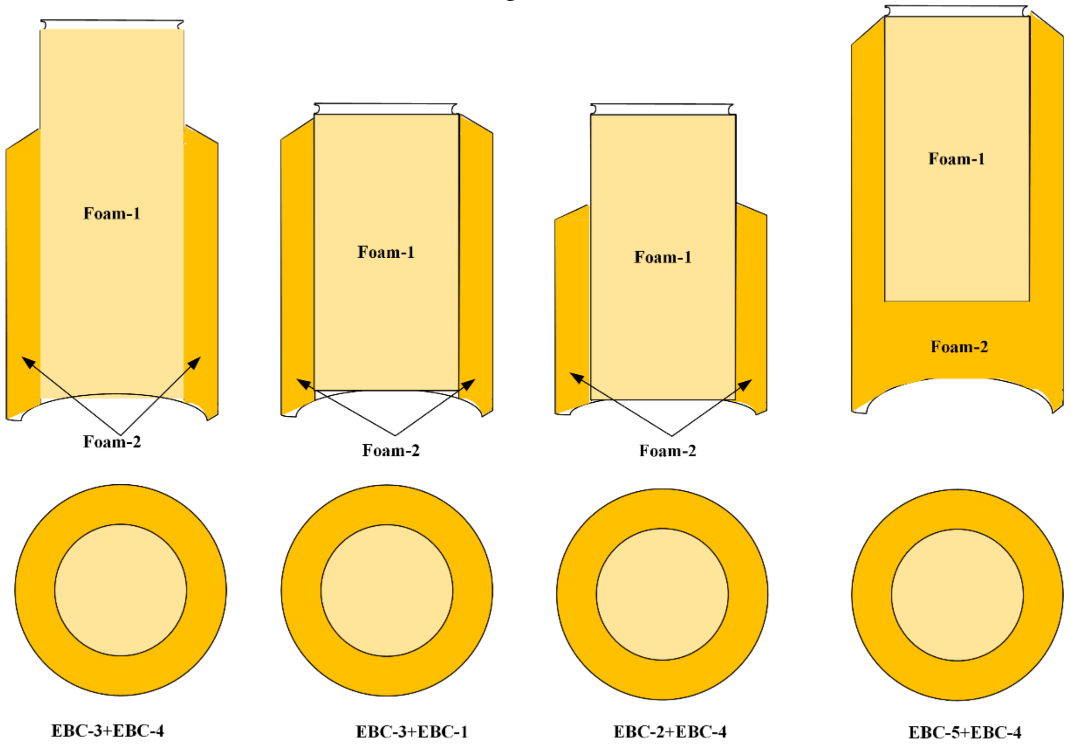

Figure S1. The style of combined configuration.

Our study follows the naming convention FFC-A, B-C, D. Here, A represents the model of the external can, with possible values of 2, 3, or 5. B denotes the model of the internal beverage can, with values 1 or 4 based on size parameters. C stands for the density between the inner and outer walls of the two cans, with D representing the density within the inner can. Currently, we are focusing on the combination of two cans, but there is potential for further research on additional combinations. Our design includes varying the can combination and the density of

the foam filling. By utilizing a combination of cans, we can create a group of concentric cylinders with different heights. The foam filling results in a sandwich structure, enhancing the axial and lateral energy absorption capacities of the structure. This setup also addresses issues such as Euler buckling in FFC-4 and the limited crushing stroke in FFC-3. To test our hypothesis, we combined EBC-3 and EBC-4 tanks, prepared two sample sets (FFC-3.4-100,40 and FFC-3.4-100,100), and conducted crush tests at a speed of 5mm/min.

Eight equidistant notches were cut into the neck of EBC-3 along the circumferential direction to allow the can body of EBC-4 to smoothly pass through the narrow position of the can mouth. This design not only aids in assembly but also helps reduce force fluctuations.

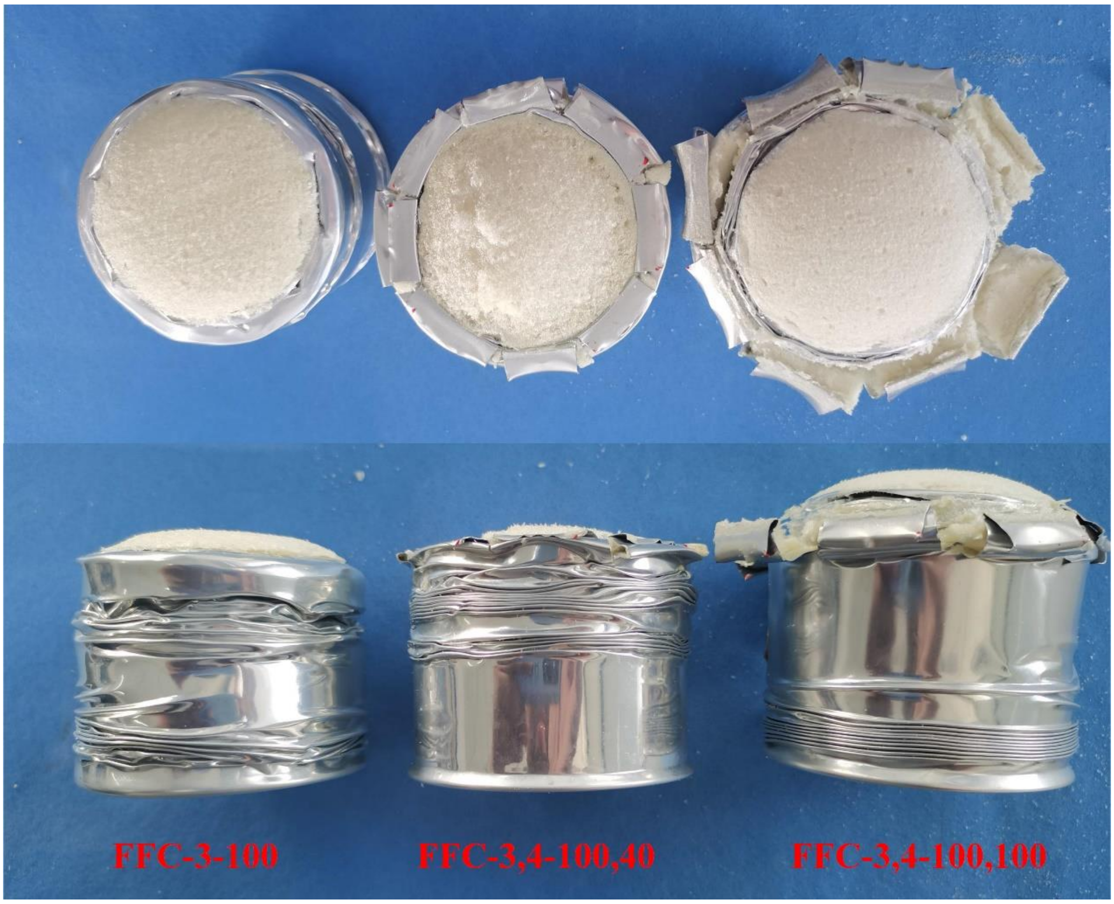

Figure S2. Crushing of different structures.

Figure S3 displays the force-displacement dashed lines and energy absorption parameters of both the combined structure and the original structure. The energy absorption and specific energy absorption of the combined mechanisms outperform those of the original structure as the foam density varies. Additionally, enhancing the foam density of the inner tube leads to improvements in the overall structure's EA and SEA.

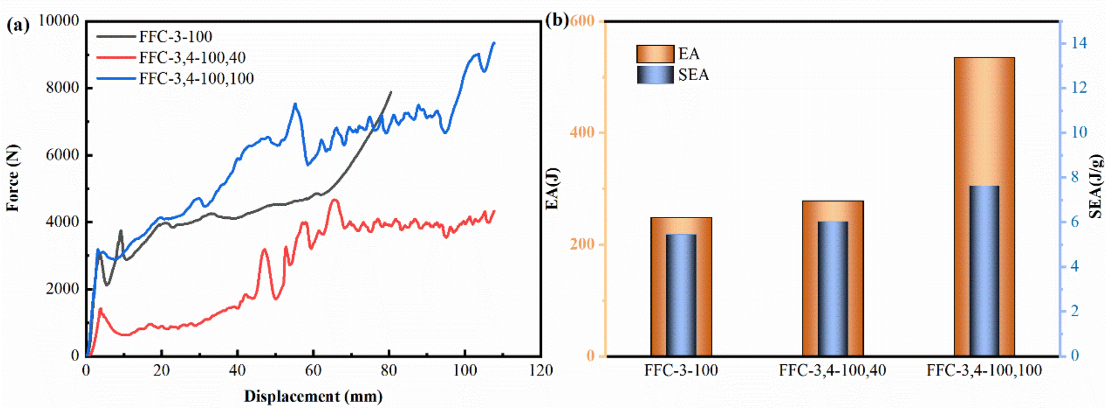

Figure S3. Energy absorption in combined and original structures. (**a**) Force-displacement curves; (**b**) EA and SEA.
